# Current Trends and Future Directions of Digital Pathology and Artificial Intelligence in Dermatopathology: A Scientometric-Based Review

**DOI:** 10.3390/diagnostics15172196

**Published:** 2025-08-29

**Authors:** Iuliu Gabriel Cocuz, Raluca Niculescu, Maria-Cătălina Popelea, Maria Elena Cocuz, Adrian-Horațiu Sabău, Andreea-Cătălina Tinca, Andreea Raluca Cozac-Szoke, Diana Maria Chiorean, Corina Eugenia Budin, Ovidiu Simion Cotoi

**Affiliations:** 1 Pathophysiology Department, “George Emil Palade” University of Medicine, Pharmacy, Sciences and Technology of Targu Mures, 540142 Targu Mures, Romania; 2 Pathology Department, Mures Clinical County Hospital, 540011 Targu Mures, Romania; 3 Histology Department, “George Emil Palade” University of Medicine, Pharmacy, Sciences and Technology of Targu Mures, 540142 Targu Mures, Romania; 4 Fundamental Prophylactic and Clinical Disciplines Department, Faculty of Medicine, Transilvania University of Brasov, 500003 Brasov, Romania; 5 Clinical Pneumology and Infectious Diseases Hospital of Brasov, 500174 Brasov, Romania; 6 Pneumology Department, Mures Clinical County Hospital, 540011 Targu Mures, Romania

**Keywords:** digital pathology, artificial intelligence, dermatopathology, whole slide imaging, telepathology, scientometrics

## Abstract

**Background:** Digital Pathology (DP) and Artificial Intelligence (AI) have strongly developed in recent years, especially in pathology, with a high interest in dermatopathology. Accelerated by the COVID-19 pandemic, DP and AI are now integrated in pathology, research and education, bringing value to histopathological diagnoses, telepathology and personalized medicine. This narrative review presents a comprehensive literature review by defining three research directions, using scientometric analysis, of the current state of DP and AI in pathology and dermatopathology. **Methods:** The research was conducted through the Pubmed and Web of Science databases, within the research period of January 2019–July 2025: a two-phase methodology. Four independent pathologists selected the articles in accordance with the inclusion and exclusion criteria, and the synthesis of the articles was based on three research directions. **Results:** The research shows that CNN (Convolutional Neural Network), AI powered diagnostic platforms and telepathology strongly contribute to increasing the speed and accuracy of diagnostics, especially on cutaneous malignant skin tumors. There are still several challenges and limitations in terms of validation, interoperability, initial high implementation costs, ethics and transparency in AI and equity in healthcare. **Conclusions:** DP and AI are essential pillars of modern dermatopathology, with a high necessity of standardization, regulation and a multidisciplinary approach.

## 1. Introduction

The last decade has been a hallmark for digital transformation in many fields, including healthcare. Pathology is one of the healthcare domains in which digital transformation, by developing Digital Pathology (DP) and Artificial Intelligence (AI) and integrating them into histopathological diagnoses, has undergone tremendous development. What was once considered innovative or experimental is nowadays integrated into daily clinical practice, research or teaching [1,2,3,4]. The accuracy of histopathological diagnoses, via the combination of Whole Slide Imaging (WSI), computational pathology and automated learning algorithms has increased alongside the development of technology. It is not only about the diagnoses, but also the redefinition of personalized medicine, with a strong focus on classifying aggressive cutaneous tumors, such as melanoma [5,6,7,8].

AI in healthcare has many applications, such as radiology, pathology and pharmacology. Also, AI is a real help in infectious diseases for the rapid identification of pathogens and epidemiological monitorization. Besides this, personalized decision making via the integration of clinical, pathological, imagistic and molecular data can be carried out using AI [9].

DP has constantly evolved since the first applications of telepathology in 1960 and upon the development and integration of AI, and is starting to be a technology with a high grade of maturity, being used in more laboratories across the world [10]. As DP is constantly evolving, it can emerge in daily activities like slide archival, in-line scanning, remote consultation and telepathology, tumor boards, education and research [11].

The progress in DP and AI, especially with the use of CNNs, has evolved tremendously in recent years, especially in dermatopathology, where assisted-AI tools are helping pathologists to establish advanced and more accurate diagnoses for skin lesions [12]. Dermatopathology is not the only field of pathology in which AI is evolving. Prostate cancer can be detected and graded through AI software and further research is being conducted on its predictive and prognostic predictions [13].

The COVID-19 pandemic has worked exactly like a catalyst for developing DP [14]. With an increased lack of access to medical services for patients, social distance regulations and the necessity of readapting all workflows, the COVID-19 pandemic accelerated the development of digital services. Because of this, DP became an essential component of maintaining the quality of histopathological diagnostics, opening the doors to worldwide medical expertise [15,16,17]. In parallel, AI has rapidly developed, with capacities of interpreting WSI and identifying regions of interest (ROI) and the ability to generate preliminary histopathological reports, with a high interest in dermatopathology [18,19].

The aim of this review is to provide a comprehensive analysis of the current state of DP and AI in dermatopathology, with a highlight on technological achievements, clinical implications, regulatory advice, perspectives and limitations in implementing DP and AI in dermatopathology through a scientometric approach, and a definition of the most recent research directions in the field. By using this approach, this review is the first of its kind in the literature at the moment of submission.

## 2. Methodology

The present review is a narrative review based on comprehensive literature research in two major biomedical databases (Web of Science and Pubmed) for articles and reviews regarding DP and AI in dermatopathology. The research process was performed in March–July 2025 and included articles published between January 2019 and July 2025. The methodology used for the review was divided into two phases.

**Figure 1:**
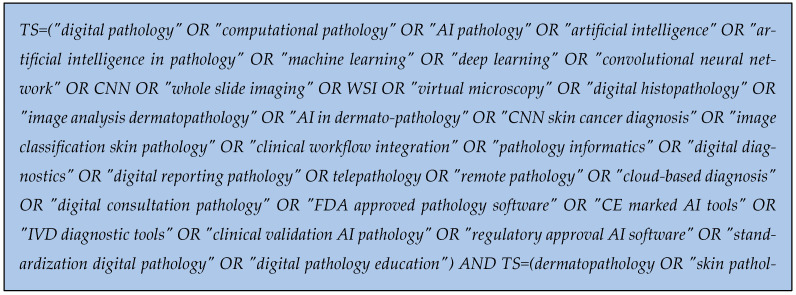
Research query for Web of Science database.

Phase one was conducted through a scientometric study on Web of Science to establish the research directions. The research started with the research query for interrogation of the Web of Science database presented in Figure 1.

**Figure 2:**
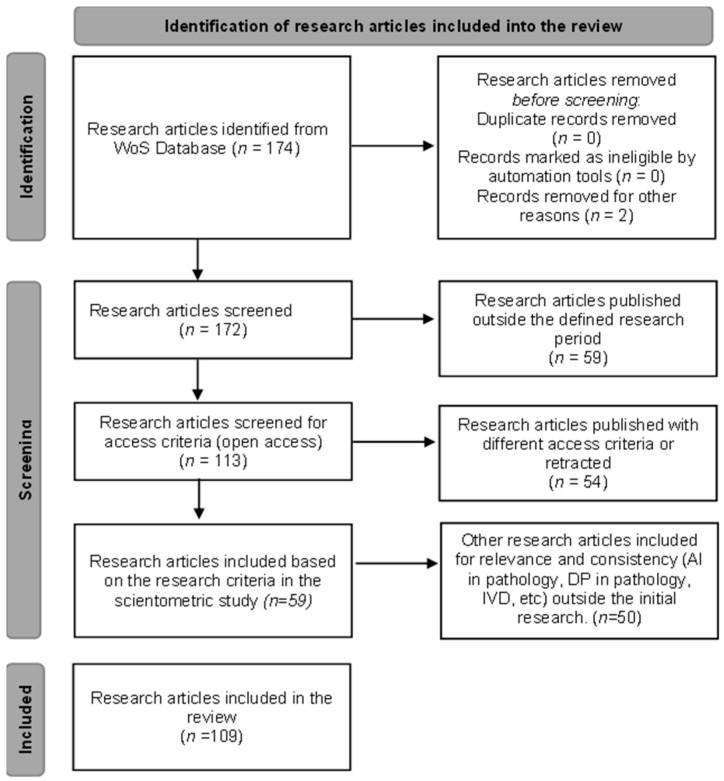
Diagram for inclusion criteria of articles in the review.

After the research was performed through Web of Science, inclusion and exclusion criteria were used to include articles in the defining research direction process as shown in the Figure 2. Beside the included articles from the research performed, several articles were included for the relevance of the review (AI in pathology, DP in pathology, IVD, etc.) from other databases (Figure 2).

**Figure 3:**
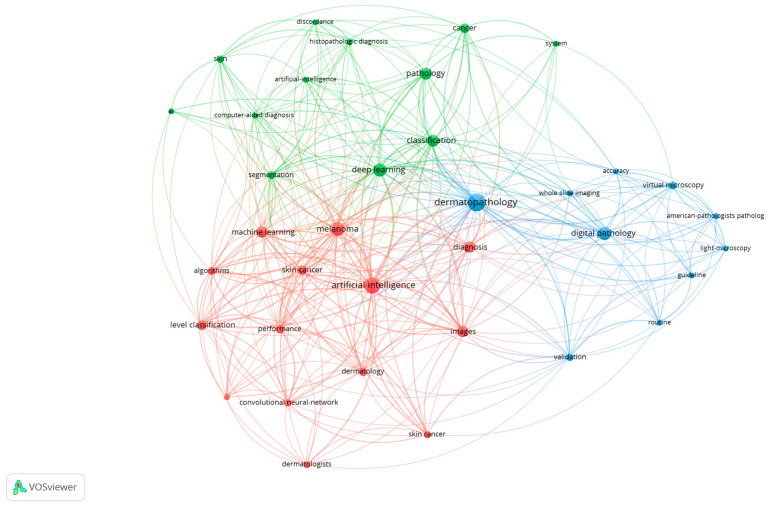
Scientometric map generated through VOSviewer using the keywords from selected articles. Cluster 1—red, Cluster 2—green, Cluster 3—blue.

**Figure 4:**
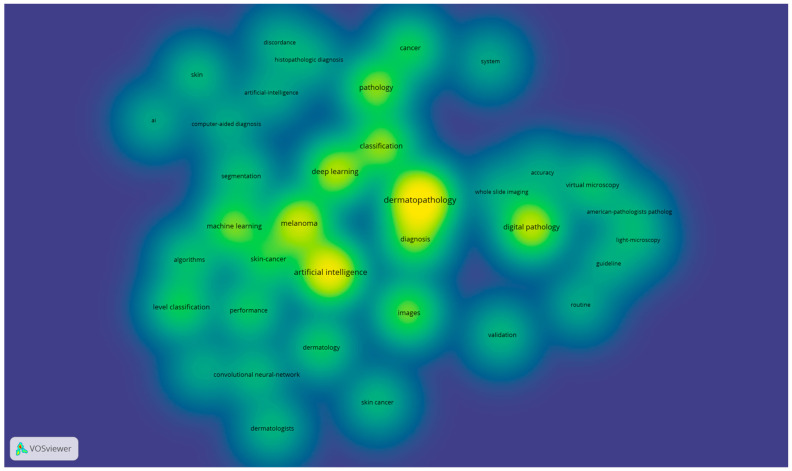
Density map generated through VOSviewer using the keywords from selected articles.

**Figure 5:**
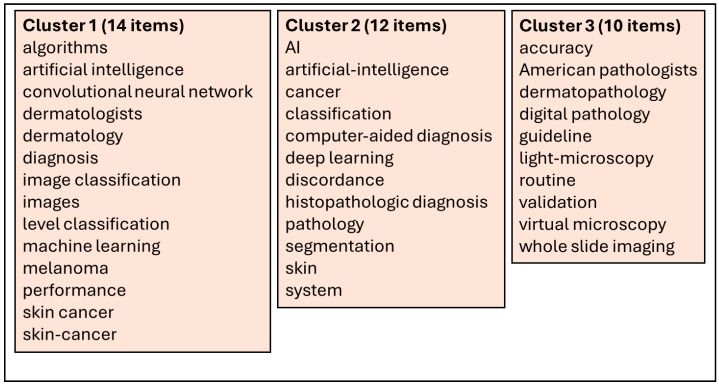
Generated clusters of keywords through VOSviewer.

Through the extracted 59 articles from the Web of Science database [4,12,20,21,22,23,24,25,26,27,28,29,30,31,32,33,34,35,36,37,38,39,40,41,42,43,44,45,46,47,48,49,50,51,52,53,54,55,56,57,58,59,60,61,62,63,64,65,66,67,68,69,70,71,72,73,74,75], we have performed a scientometric analysis based on the keywords used in the abstract of every article. For this, we used the VOSviewer software (version 1.6.20, released on 31 October 2023) [76] to extract the keywords from each article, the relations between them and to generate the scientometric maps and the density maps. A total of 289 keywords were included in the scientometric analysis, with a minimum threshold of three occurrences of the keywords in the selected articles. Using this threshold, a total of 36 keywords were included for the scientometric cartographies based on their co-occurrences and the total link strength. After processing through VOSviewer, based on the scientometric map generated (Figure 3) and the density map of keywords (Figure 4), three clusters of keywords were generated based on co-occurrences and connections between them, as shown in Figure 5.

**Figure 6:**
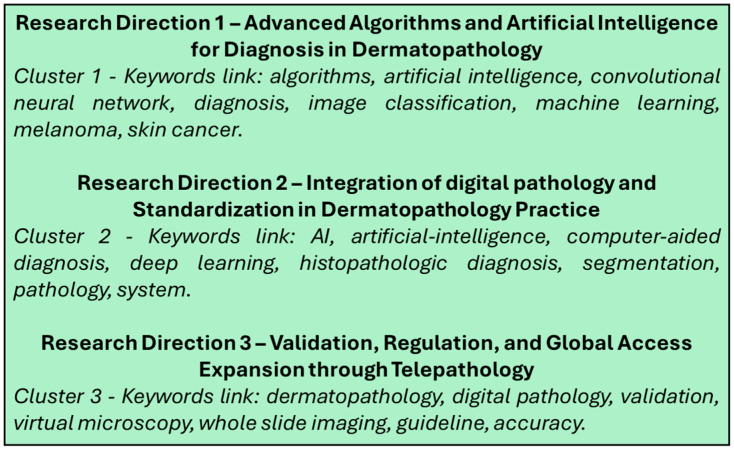
Research directions generation by clusters of keywords.

Based on these three clusters we established the three research directions for digital pathology and artificial intelligence in dermatopathology, as shown in Figure 6.

Phase two was the review of the articles included in the study. As shown in Figure 2, besides the articles included to establish the research directions, several articles were included for consistency and relevance. The selection of articles was made by four independent reviewers (pathologists) who underwent a screening process for all articles. A total of 109 articles met all the inclusion criteria and were included in the study. No patients or patient data were used for this review. 

## 3. Research Direction 1—Advanced Algorithms and Artificial Intelligence for Diagnosis in Dermatopathology

**Figure 7:**
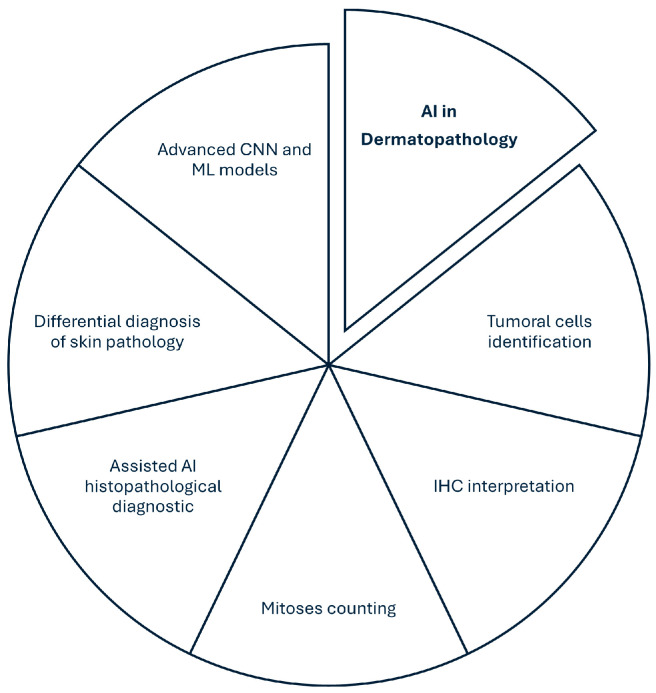
AI usage in dermatopathology [20,26,29,31,33,42,45,47,50,51,67].

Artificial intelligence is nowadays considered by pathologists to be a valuable partner. As AI is continuously developing, AI algorithms can pre-screen the slides, highlighting the interest zones or even providing a preliminary interpretation (Figure 7). AI is already being used in dermatopathology for the classification of tumoral lesions and the detection of melanocytic pathologies [77,78].

Artificial intelligence (AI) has developed rapidly and is emerging in every field, with a special interest in healthcare. In pathology, AI is allowing for the integration of histopathological, molecular and clinical data, resulting in a strong point for personalized medicine by identifying therapeutical biomarkers and establishing a degree of risk classification, including aggressive tumors in dermatopathology, like melanoma [77].

The integration of AI into different platforms and cloud systems may represent a real help and offer diagnostic services in regions where there is a lack of dermatopathologists, and by this, contribute to the decrease in imbalance in healthcare equity throughout the world [48,79].

By integrating AI into dermatopathology, a series of transversal benefits like stratification and diagnostic accuracy, prediction of treatment response and medical education may be the source of revolution in the domain [77]. The integration of AI into dermatopathology has the support of the worldwide medical community, with a high percentage of consensus for increasing diagnostic efficiency and accuracy, with 84.1% of dermatopathology specialists believing that AI should be included in medical education [26]. Progress in integrating AI depends on a multidisciplinary approach, with strong collaboration between dermatologists, other clinicians, bioinformatics and engineers. An important aspect is the importance that is given to the doctor–patient relationship and the decision-making responsibilities that can be adjusted with AI [29,51,80].

Emerging AI technologies, like Generative AI (which can generate rare histopathological images) and federated learning (which may train models without affecting the confidentiality of the data) go beyond the educational and diagnostic roles of AI, increasing the confidentiality levels and reducing ethical concerns, therefore increasing the quality of generative models [77].

Standardization is the most important fact that needs to be taken into consideration while advancing AI in dermatopathology. Firstly, the automatization of repetitive tasks, such as excision margins evaluation or the mitotic figures count can be implemented to standardize the process and further concentrate the resources of the lab for more complex cases [77]. Optimization and standardization through the reporting system must be taken into consideration. Multiple AI software, like ReportTutor [81], use a natural language model to help doctors to generate diagnostic reports which are in accordance with reality, promoting standardization into medical practice. HistoGPT (Helmholtz Munich, 2025, Germany) [82] is also a generative AI integration tool for generating pathology reports from images and thereby leading to future standardization.

Deep learning algorithms, especially convolutional neural networks (CNNs) have demonstrated an accuracy of 95% in diagnostics in dermatopathology [48,77,78,83]. CNNs can now be applied to WSI and thereby process digital images, with a component of assisted or independent diagnostics, reducing the time needed for dermatopathology diagnostics. Interobserver variability may be reduced when diagnosing basal cell carcinoma or melanoma. Machine learning (ML) algorithms like Fast Random Forest can also reduce interobserver variability, for example regarding the differences between dysplastic nevi and melanoma [29].

ResNet and VGG-19 (Visual Geometry Group, University of Oxford, 2014, Oxford, UK) models which were trained on over 9.95 million histological patches from WSI, have demonstrated a superior accuracy in differentiating melanocytic nevi from melanoma [80,84]. PDLS (Pathology Deep Learning Systems) are using CNNs on multiple levels like image adaptation, identifying the region of interest and final classification with trust scores. This multistep approach is increasing the confidence level in comparison to classic approaches [20]. AutoML can facilitate the integration of AI models without advanced technical knowledge and allows doctors to implement models directly in their electronic medical record systems (EMR) [29,83].

Another direct usage of AI in dermatopathology is tumor classification support, in which, using morphological characteristics, AI can distinguish between many tumor types like basal cell carcinoma (BCC) and cutaneous squamous cell carcinoma (cSCC) [84].

A study performed on 386 cases of basal cell carcinoma (BCC), cutaneous squamous cell carcinoma (cSCC), nevi, melanoma and other non-tumoral tissue with a result on WSI of 129,364 patches using the EfficientNetV2-S model revealed an accuracy of 98.7% for tumoral cases, with a confusion between melanoma and BCC-cSCC [31].

A study that created an end-to-end deep learning framework for skin tumors (neurofibroma, Bowen Disease and seborrheic keratosis) used two different approaches: a patch-wise classification by dividing each WSI image into patches and classifying them using different CNNs—the most high-performing being EfficientNetB6—and a classification by slide-wise aggregation, by combining the characteristics of the patches with an Attention Graph Gated Network (AGGN). The multitude of CNNs used (Xception, ResNet50, InceptionV3, Dual Path Network, EfficientNetB6), the optimization and the heatmaps used for interpretation improved the consistency of the developed model in relation to the pathologist [26].

The algorithms used for counting the mitoses were developed initially for breast carcinoma but applied also to melanocytic cutaneous lesions. A study performed on 99 cases using a CNN-based mitosis algorithm with a manual selection of ROIs detected 2868 candidate objects. After selection, there were 825 candidate mitoses identified in 61 cases (lesional or non-lesional), but with a high rate of false positive results (melanin pigment, other nuclei or artefacts). One advantage is that the algorithm can identify mitoses which are hard to observe, especially in atypical melanocytic lesions. There is still a need for fine-tuning for the algorithm to be able to make a distinction between melanocytic and non-melanocytic mitoses [50].

Another application is the screening and diagnostics of nevoid melanoma. The first use of Fast Random Forest for dermatopathology pre-screening was on two classification clusters of pixels on WSI (C1—normal clusters and C2—possible anormal clusters—nevoid melanoma) [47]. Cutaneous lymphomas could use advanced AI for detection and triage, subclassification depending on rare entities, identification or quantification of biomarkers and the prediction of prognostics and treatment response. There is a possibility of using other validated AI algorithms (melanoma, basal cell carcinoma) on cutaneous lymphoma for further validation [45].

The differential diagnosis of Melanoma Nodal Metastasis (NM) and Intra-Nodal Nevus (INN) was assessed using an AI algorithm on 196 sentinel node biopsies with a total of 485 WSI. There were 5956 annotated pixel-wise regions guided by immunohistochemistry. A CNN model was proposed with a U-NET architecture and two layers (parent-child). The architecture was designed on two layers: layer one (parent)—detection of quality tissue with the elimination of artefacts and layer two (child)—the detection of NM and INN in the zones validated by layer one. NM had a sensibility of 89% and a specificity of 94%, while INN had a sensibility of 78% and a specificity of 63%. The AI model also detected small tumoral foci, missed by the pathologist. The main advantage was the double detection (NM+INN) with a reduced rate of immunohistochemistry dependence, by training the algorithm with the morphological characteristics on H&E slides [33].

The UNet CNN model was used also to detect the clear excision margins in BCC by the use of both the classic UNet model and the UNet with ResNet34 encoder, also using deep supervision on 650 WSI of BCC and 3443 tissue sections with an accuracy of detection of 96.4%, a sensibility of 96.3% and a specificity of 36.5% [43].

A factor of hesitation for the implementation of AI in pathology is still represented by the black-box risks, e.g., the lack of transparency or the possibility of visualizing the decisional thinking of the algorithm. By implementing explicable AI technology (XAI) this hesitation might be reduced. Legal responsibility in cases of errors generated by AI is still a concern, and XAI may mitigate this concern by providing transparency in decision-making [20,85].

Even though AI evolves and new models are developing in real time, adaptive AI, in which AI models that adapt in real-time with new cases, raises questions in terms of the reproducibility of cases but also of responsibility in case of errors. Like in every field, in pathology, errors in the oncologic diagnostic may lead to cataclysmic consequences, therefore there is a need for adequate technical regulations [26,29,86].

Adaptative learning AI and the risk of “black-box” AI, regarding the lack of transparency in the decisional process of the AI algorithm may lead to a lack of low trustworthiness and difficulties in legal traceability, but may also lead to reproducibility concerns [20,85]. Table 1 integrates the main roles of AI in dermatopathology at the time of the study.

## 4. Research Direction 2—Integration of Digital Pathology and Standardization in Dermatopathology Practice

DP is no longer considered experimental or merely innovative and nowadays has reached a high degree of maturity and is implemented in hospitals, clinical centers, various pathology services and many academic institutions [19]. Digital infrastructure combined with whole slide imaging (WSI) has led to the transition from optical microscopy to complete digital analysis, even for the primary diagnosis. DP has a great utility in dermatopathology, where the complexity of the lesions and the histopathological variability may need a second opinion [84]. Besides this, combining WSI with molecular pathology and clinical data, DP and AI may lead to the early detection of aggressive tumors like melanoma, thereby increasing the quality of therapeutic decisions and the overall survival of the patients [77].

Because we live in the era of digitalization, many pathology services have totally transformed their workflow into a completely digital one. The implementation of WSI scanners on a large-scale basis, with cloud infrastructure and standardized international software for interpretation are the essential conditions to establish a well-organized telepathology service. The rapid evolution of high-speed internet access and the development of cloud systems is facilitating international collaboration. Digital infrastructure with high levels of cybersecurity is reinforcing the feasibility of telepathology [87,88].

The integration of DP into dermatopathology also has two important results: increasing the quality of medical education and supporting research and innovation. The teaching processes in histology, pathology and cellular biology have been reinterpreted by the integration of DP. Cases can be annotated, archived and very easily shared and the evaluation can be easily standardized. In dermatopathology, digital collections of cutaneous lesions may lead to superior visual training [48,83]. Research in dermatopathology may also be increased by using digital libraries of WSI which are structured and standardized, therefore helping computational pathology [48]. These represent the foundations for developing AI algorithms, retrospective studies, metanalyses and international collaborations in oncology and pathology.

DP and AI in dermatopathology have made significant progress in the last years, but as always, high development comes with significant limitations. The lack of interoperability between different DP and AI systems is one of the major limitations which may affect the standardization of the diagnostic process. Regarding cost, the implementation of DP and AI in pathology and dermatopathology incurs high initial costs, which is a major impediment for many medical centers [2,3,48,84,89]. Validation may raise concerns in each laboratory, even if the software or device is FDA- or IVD-approved, in terms of local validation, which may lead to delays in implementation [67,89,90,91,92].

Studies across pathologists shows that there exists professional support to implement DP in laboratories, especially for standardizable duties like the identification of mitoses, tumoral excision margins or immunohistochemical evaluation. Also, the standardization of WSI infrastructures and workflows is needed for global interoperability [49].

Table 2 integrates the main roles of digital pathology in dermatopathology at the time of the study.

## 5. Research Direction 3—Validation, Regulation, and Global Access Expansion Through Telepathology

Validation depends on various factors and should be classified as technical validation, in which, starting from the structure of the software, the developing code, the composition of the algorithm, user interface, and total performance all are technical components, and clinical validation, which always comes after technical validation, in which the developed software must comply to the impact of direct usage for diagnostics, especially in dermatopathology, where there might often be a difference between cancer or no cancer. This means that clinical validation relies mostly on the medical decision that is performed by the AI software [45,46].

As more laboratories are in a process of transition between classic pathology and digital pathology, this opens the possibility for real-life testing in many laboratories, ensuring multi-step testing in many different pathology services, with different workflows and different environments. Even though software receives the certification of an IVD software, validation goes further than the company that releases the software. Therefore, local validation (or third validation in the process) should be taken into consideration, before applying the AI software or telepathology software in the standard workflow of the pathology service. Validation should always be taken into consideration when testing AI software in terms of pathology, as each laboratory may use different devices, different scanners for WSI and different computers. Heterogeneity in testing is always necessary for approval by the FDA or the EC, and for the internationalization of the process [67,89,92,93,94].

AI software which is used in diagnostic procedures must be validated as medical device software and must be distinguished from research-use-only (RUO) software. For this, worldwide acceptance of AI software must be authorized by the authorities (the FDA in the USA or the European Commission (EC) in Europe) as medical software, with complete compliance with MDR and IVD recognition. Besides those accreditations, ISO protocols for medical devices which come with supplementary indications should be taken into consideration [89,92].

As of 2025, the FDA has approved almost 1000 AI-based medical devices, but the regulation of AI must be flexible and adapted to the rapid rhythm of development, coordinated through governmental agencies. Developers must maintain a high transparency of the AI algorithms and the results of developing an AI medical tool must be determined by the patient’s health, not only the operational or economic benefits [95]. A study performed on IVD validation showed that out of the 26 AI products identified for DP on the EEA/GB market, 24 were IVD-approved by auto-evaluation. Only 38% of the products had internal validation studies and 42% had external studies. An online registry for AI products of DP was developed (Register of AI products for digital pathology—https://osf.io/gb84r/ (accessed on 14 August 2025)) [89]

AI models designed for pathology, besides validation, need to be carefully documented. Every stage in production, development and validation should be precisely documented and this documentation should be supported by accredited institutions. In addition, for the software to be used in clinical practice, the algorithm needs to be completely explicable (interpretable AI). To serve as interoperable software, standardization needs to be implemented in terms of images and clinical data transfer and analyzing, such as DICOM for images and mostly HL7 for clinical data. This is a necessity and an obligation to be able to receive the accreditation for clinical usage. The risk assessment is strictly regulated by IVD and FDA regulations, and for this each software should be classified or evaluated through the risk-based classification of the IVD and FDA regulations [67,89,93,94,95,103,104,105].

In the absence of a structured ethical framework, AI may amplify the existing inequalities in dermatopathology. Without adequate control, AI might become an instrument that highlights the disparities between more developed medical centers and those which are not so developed. AI models can also acquire information from images provided through the optic tube of the microscope, representing a way of implementing DP and AI in services with limited resources [96,97,98]. The fact that there is not an ethical framework regarding AI in dermatopathology, the risk of exacerbating existing healthcare disparities in healthcare centers may become very significant, combined with the legal responsibility in case of a medical error. All of these limitations appear when the world evolves and technology gets better [29,48,79,106].

Cybersecurity nowadays is a concern and the implementation of very strict and serious regulations regarding medical data in every healthcare facility is necessary, especially in terms of telepathology and cloud-based systems, with GDPR/HIPAA regulations to be respected [19,77,78,84,90]. By focusing on continuous improvement, DP in dermatopathology will be improved in the future.

The integration of DP has faced many technical troubles in terms of standardization and infrastructure. A big limitation is the absence of interoperability between various systems, the necessity of very accurate and modern servers, the initial high costs for implementation and the need for standardized protocols for cybersecurity and archiving. International standards for medical pictures, such as DICOM, are rapidly evolving and widely accepted for WSI [2,99,100].

In terms of de facto usage in a laboratory, each process that involves quality assurance and control should be reconsidered when the transition is made to AI pathology software and DP implementation, regarding the pre-analytical, analytical and post-analytical process. To assure quality control, all personnel of the laboratory should be carefully instructed on how to use the devices or the software, and the FDA and the MDR should request the documented instructions of the personnel who will work with or use the validated software [67,93,94,103,104,105].

The future of telepathology depends on the development of collaborative medicine where dermatopathologists, dermatologists, surgeons, oncologists and molecular pathologists from other services construct the framework for an integrated therapeutical decision-making model [87,88,107]. The relevance of clinical studies in dermatopathology and the statistical power of multicentric networks are increased by telepathology, in terms of the development of new AI algorithms, the validation of predictive and diagnostics biomarkers and the evaluation of diagnostic interobserver variances [101,102]. Personalized medicine for melanoma will benefit from a holistic approach, in which histopathological examination needs to be contextualized in a clinical and molecular pathway [87,88,101,102].

Telepathology can be considered the branch of DP that brings together the transmission, visualization and interpretation from distance of the histopathological images from microscopic slides through communication systems. This allows for the establishment of the histopathological diagnosis without the physical presence of a pathologist. Telepathology can be classified as static, dynamic and hybrid, depending on the amount of control that involves a physical presence [40,107].

The progress in DP and WSI nowadays allows for a diagnosis in dermatopathology, and a second opinion without the physical movement of the samples. By this, it can be said that it can be a global framework for dermatopathology worldwide. Telepathology is a great way of optimizing human resources within a pathological service. AI algorithms can pre-classify routine cases and focus on those which may need extensive attention or a second opinion. Worldwide, there are pathology services which do not have specialized dermatopathologists. Telepathology is a real help in increasing the healthcare services provided by that service, in terms of using it to provide urgent diagnoses and avoid delays in the treatment of a malignant skin lesion [87,88,101,102].

An important point in developing telepathology is represented by the necessity of following GDPR rules (in Europe) and HIPAA (in USA). Cybersecurity remains a problem in terms of medical data, and a threat. Without establishing serious real-time data protection protocols, access control and audits for veracity, trust in DP and telepathology may be affected [19,78,84,90,91].

## 6. Conclusions, Foundations and Challenges of Implementing DP and AI in Dermatopathology

DP has now reached a level of maturity in which we can say that it is no longer the future, it is the present. With the variety of domains in which DP can be implemented, such as pathology services, hospitals, research centers and academic institutions, we can say that the use of DP and AI in dermatopathology has evolved very rapidly. DP is valuable in terms of the early diagnosis of aggressive cutaneous tumors such as melanoma, by integrating histopathological data, clinical data and molecular data, and thereby being able to increase the quality of medical activities, increasing the quality of life for the patients. Even though the COVID-19 pandemic has been a challenge worldwide, it can be said that in terms of developing DP, especially in dermatopathology, the pandemic forced the technology to develop and telepathology to evolve. DP and AI are already being applied in dermatopathology and being used with a high grade of accuracy in terms of tumor classification and distinguishing malignant cases from benign ones. DP also supports continuous medical teaching and research, enhancing the quality of teaching using new and advanced technologies. Standardization brings together multicenter databases and approaches through DP. The ethics and confidentiality of medical data are strongly improved by generative AI and federated learning, increasing trust regarding implementing AI into daily routine clinical practices. The development of CNN algorithms comes with an increased percentage of accuracy in histopathological diagnostic sand with telepathology that brings in a worldwide perspective, the quality of histopathological diagnostics is increasing. Multistep validation is necessary and remains the biggest challenge for the implementation of AI and DP in daily routines.

## Figures and Tables

**Table 1:** Main applications of AI in dermatopathology.

Reference	Methodology/Technology	Application/Usage
[20,29,51,77]	AI pre-screening, CNNs	Highlighting regions of interest, preliminary slide interpretation, tumor lesion classification.
[26]	Attention Graph Gated Network + EfficientNetB6	End-to-end DL framework for multiple skin tumors; patch-wise and slide-wise classification.
[29,83]	AutoML	Integration of AI in EMR systems by clinicians without technical expertise.
[31]	EfficientNetV2-S	WSI of 386 skin tumors; 98.7% accuracy, confusion between melanoma and BCC/cSCC.
[33]	CNN (U-Net, parent–child layers)	Differentiation of nodal metastasis (NM) vs. intranodal nevus (INN); high sensitivity/specificity.
[45]	AI for cutaneous lymphoma	Subclassification, biomarker identification, prognostic prediction.
[47]	Fast Random Forest	Pre-screening nevoid melanoma on WSI pixel clusters.
[80,84]	CNNs	WSI classification of melanocytic nevi vs. melanoma; ~95% accuracy.
[50]	CNN-based mitosis detection	Applied to melanocytic lesions; improved mitosis identification but false positives.
[80,84]	ResNet (Microsoft), VGG-19 (Oxford)	>9.9 M histology patches; melanoma vs. nevi classification with high accuracy.
[81]	ReportTutor (NLP model)	Automated report generation, promoting standardization.
[82]	HistoGPT (Generative AI)	Generating pathology reports/images, aiding education and diagnostics.
[85]	Explainable AI (XAI)	Improving transparency, mitigating “black-box” risks in clinical adoption.

**Table 2:** Main applications based on digital pathology in dermatopathology.

Reference	Methodology/Technology	Application/Usage
[19]	Whole Slide Imaging (WSI), telepathology	Transition from optical to digital microscopy; secure cloud infrastructure for primary diagnosis.
[48,83]	Digital collections and archives	Education, annotation, standardization, dermatopathology teaching.
[67,89,90,91,92]	Validation studies, IVD software	Local and international validation of DP platforms; regulatory approval concerns.
[84]	Integration of WSI with molecular data	Early detection of aggressive melanoma; therapeutic decision-making.
[87,88]	Cloud systems + telepathology	International collaboration, remote consultation, second opinions, optimized human resources.
[89,92,93,94,95]	FDA/CE regulation and ISO protocols	Classification of DP/AI as medical devices; risk-based validation.
[89]	Registry of AI/DP products (Europe)	Tracking validation and certification of AI-based DP software.
[96,97,98]	Low-cost DP (microscope camera, cloud)	Implementation in resource-limited settings.
[99,100]	DICOM standards for pathology	Standardization and interoperability for WSI images.
[101,102]	Telepathology in collaborative networks	Multicenter research, biomarker validation, second-opinion services.

## Data Availability

The data presented into this review is publicly available on the databases specified in the methodology.

## Abbreviations

The following abbreviations are used in this manuscript: DP (Digital Pathology), AI (Artificial Intelligence), WSI (Whole Slide Imaging), ROI (Region of Interest), CNN (Convolutional Neural Network), RNN (Recurrent Neural Network), PDLS (Pathology Deep Learning Systems), EMR (Electronic Medical Record), XAI (Explainable Artificial Intelligence), GDPR (General Data Protection Regulation), HIPAA (Health Insurance Portability and Accountability Act), FDA (Food and Drug Administration), EC (European Commission), IVD (In Vitro Diagnostic), MDR (Medical Device Regulation), ISO (International Organization for Standardization), DICOM (Digital Imaging and Communications in Medicine), HL7 (Health Level Seven International), RUO (Research Use Only), ML (Machine Learning), CE (Conformité Européenne—European Conformity).

## References

[1] Bruce C., Prassas I., Mokhtar M., Clarke B., Youssef E., Wang C., Yousef G.M. (2024). Transforming Diagnostics: The Implementation of Digital Pathology in Clinical Laboratories. Histopathology.

[2] Betmouni S. (2021). Diagnostic Digital Pathology Implementation: Learning from the Digital Health Experience. Digit. Health.

[3] Jahn S.W., Plass M., Moinfar F. (2020). Digital Pathology: Advantages, Limitations and Emerging Perspectives. J. Clin. Med..

[4] Hanna M.G., Reuter V.E., Hameed M.R., Tan L.K., Chiang S., Sigel C., Hollmann T., Giri D., Samboy J., Moradel C. (2019). Whole Slide Imaging Equivalency and Efficiency Study: Experience at a Large Academic Center. Mod. Pathol..

[5] Jansen P., Baguer D.O., Duschner N., Arrastia J.L., Schmidt M., Landsberg J., Wenzel J., Schadendorf D., Hadaschik E., Maass P. (2023). Deep Learning Detection of Melanoma Metastases in Lymph Nodes. Eur. J. Cancer.

[6] Chatziioannou E., Roßner J., Aung T.N., Rimm D.L., Niessner H., Keim U., Serna-Higuita L.M., Bonzheim I., Cuellar L.K., Westphal D. (2023). Deep Learning-Based Scoring of Tumour-Infiltrating Lymphocytes Is Prognostic in Primary Melanoma and Predictive to PD-1 Checkpoint Inhibition in Melanoma Metastases. EBioMedicine.

[7] Fatima G., Alhmadi H., Mahdi A.A., Hadi N., Fedacko J., Magomedova A., Parvez S., Raza A.M. (2024). Transforming Diagnostics: A Comprehensive Review of Advances in Digital Pathology. Cureus.

[8] Wen Z., Wang S., Yang D., Xie Y., Chen M., Bishop J., Xiao G. (2023). Deep Learning in Digital Pathology for Personalized Treatment Plans of Cancer Patients. Semin. Diagn. Pathol..

[9] Koteluk O., Wartecki A., Mazurek S., Kołodziejczak I., Mackiewicz A. (2021). How Do Machines Learn? Artificial Intelligence as a New Era in Medicine. J. Pers. Med..

[10] Rizzo P.C., Caputo A., Maddalena E., Caldonazzi N., Girolami I., Dei Tos A.P., Scarpa A., Sbaraglia M., Brunelli M., Gobbo S. (2023). Digital Pathology World Tour. Digit. Health.

[11] Zarella M.D., Bowman D., Aeffner F., Farahani N., Xthona A., Absar S.F., Parwani A., Bui M., Hartman D.J. (2019). A Practical Guide to Whole Slide Imaging: A White Paper from the Digital Pathology Association. Arch. Pathol. Lab. Med..

[12] Cazzato G., Massaro A., Colagrande A., Lettini T., Cicco S., Parente P., Nacchiero E., Lospalluti L., Cascardi E., Giudice G. (2022). Dermatopathology of Malignant Melanoma in the Era of Artificial Intelligence: A Single Institutional Experience. Diagnostics.

[13] Marletta S., Eccher A., Martelli F.M., Santonicco N., Girolami I., Scarpa A., Pagni F., L’Imperio V., Pantanowitz L., Gobbo S. (2024). Artificial Intelligence–Based Algorithms for the Diagnosis of Prostate Cancer: A Systematic Review. Am. J. Clin. Pathol..

[14] Ahuja S., Zaheer S. (2024). Advancements in Pathology: Digital Transformation, Precision Medicine, and Beyond. J. Pathol. Inform..

[15] Yousif M., Hassell L., Pantanowitz L. (2022). Impact of COVID-19 on the Adoption of Digital Pathology. Digital Innovation for Healthcare in COVID-19 Pandemic.

[16] Lujan G.M., Savage J., Shana’ah A., Yearsley M., Thomas D., Allenby P., Otero J., Limbach A.L., Cui X., Scarl R.T. (2021). Digital Pathology Initiatives and Experience of a Large Academic Institution during the Coronavirus Disease 2019 (COVID-19) Pandemic. Arch. Pathol. Lab. Med..

[17] Hanna M.G., Reuter V.E., Ardon O., Kim D., Sirintrapun S.J., Schüffler P.J., Busam K.J., Sauter J.L., Brogi E., Tan L.K. (2020). Validation of a Digital Pathology System Including Remote Review during the COVID-19 Pandemic. Mod. Pathol..

[18] Browning L., Colling R., Rakha E., Rajpoot N., Rittscher J., James J.A., Salto-Tellez M., Snead D.R.J., Verrill C. (2020). Digital Pathology and Artificial Intelligence Will Be Key to Supporting Clinical and Academic Cellular Pathology through COVID-19 and Future Crises: The PathLAKE Consortium Perspective. J. Clin. Pathol..

[19] McGenity C., Clarke E.L., Jennings C., Matthews G., Cartlidge C., Freduah-Agyemang H., Stocken D.D., Treanor D. (2024). Artificial Intelligence in Digital Pathology: A Systematic Review and Meta-Analysis of Diagnostic Test Accuracy. npj Digit. Med..

[20] Wells A., Patel S., Lee J.B., Motaparthi K. (2021). Artificial Intelligence in Dermatopathology: Diagnosis, Education, and Research. J. Cutan. Pathol..

[21] Jartarkar S.R. (2023). Artificial Intelligence: Its Role in Dermatopathology. Indian J. Dermatol. Venereol. Leprol..

[22] Wei M.L., Tada M., So A., Torres R. (2024). Artificial Intelligence and Skin Cancer. Front. Med..

[23] Young A.T., Xiong M., Pfau J., Keiser M.J., Wei M.L. (2020). Artificial Intelligence in Dermatology: A Primer. J. Investig. Dermatol..

[24] Schmitt M., Maron R.C., Hekler A., Stenzinger A., Hauschild A., Weichenthal M., Tiemann M., Krahl D., Kutzner H., Utikal J.S. (2021). Hidden Variables in Deep Learning Digital Pathology and Their Potential to Cause Batch Effects: Prediction Model Study. J. Med. Internet Res..

[25] Chan S., Reddy V., Myers B., Thibodeaux Q., Brownstone N., Liao W. (2020). Machine Learning in Dermatology: Current Applications, Opportunities, and Limitations. Dermatol. Ther..

[26] Shi Z., Zhu J., Yu L., Li X., Li J., Chen H., Chen L. (2023). A Two-Stage End-To-End Deep Learning Framework for Pathologic Examination in Skin Tumor Diagnosis. Am. J. Pathol..

[27] Sauter D., Lodde G., Nensa F., Schadendorf D., Livingstone E., Kukuk M. (2022). Validating Automatic Concept-Based Explanations for AI-Based Digital Histopathology. Sensors.

[28] Martorell A., Martin-Gorgojo A., Rios-Vinuela E., Rueda-Carnero J.M., Alfageme F., Taberner R. (2022). Artificial Intelligence in Dermatology: A Threat or an Opportunity?. Actas Dermo-Sifiliogr..

[29] Cazzato G., Rongioletti F. (2024). Artificial Intelligence in Dermatopathology: Updates, Strengths, and Challenges. Clin. Dermatol..

[30] Rezk E., Eltorki M., El-Dakhakhni W. (2022). Leveraging Artificial Intelligence to Improve the Diversity of Dermatological Skin Color Pathology: Protocol for an Algorithm Development and Validation Study. JMIR Res. Protoc..

[31] Kriegsmann K., Lobers F., Zgorzelski C., Kriegsmann J., Janssen C., Meliss R.R., Muley T., Sack U., Steinbuss G., Kriegsmann M. (2022). Deep Learning for the Detection of Anatomical Tissue Structures and Neoplasms of the Skin on Scanned Histopathological Tissue Sections. Front. Oncol..

[32] Amin S., Mori T., Itoh T. (2019). A Validation Study of Whole Slide Imaging for Primary Diagnosis of Lymphoma. Pathol. Int..

[33] Siarov J., Siarov A., Kumar D., Paoli J., Molne J., Neittaanmaki N. (2024). Deep Learning Model Shows Pathologist-Level Detection of Sentinel Node Metastasis of Melanoma and Intra-Nodal Nevi on Whole Slide Images. Front. Med..

[34] Mohammed E.J., Eliahiai I., Chaib S., Elmorabit K., Mouatakid M., Kharmoum J., Chraibi M. (2024). The State of Telepathology in Africa in the Age of Digital Pathology Advancements: A Bibliometric Analysis and Literature Review. Cureus J. Med. Sci..

[35] Chong Y., Kim D.C., Jung C.K., Kim D.-C., Song S.Y., Joo H.J., Yi S.-Y., Medical Informatics Study Group of the Korean Society of Pathologists (2020). Recommendations for Pathologic Practice Using Digital Pathology: Consensus Report of the Korean Society of Pathologists. J. Pathol. Transl. Med..

[36] Grant S.R., Andrew T.W., Alvarez E.V., Huss W.J., Paragh G. (2022). Diagnostic and Prognostic Deep Learning Applications for Histological Assessment of Cutaneous Melanoma. Cancers.

[37] Cho W.C., Gill P., Aung P.P., Gu J., Nagarajan P., Ivan D., Curry J.L., Prieto V.G., Torres-Cabala C.A. (2021). The Utility of Digital Pathology in Improving the Diagnostic Skills of Pathology Trainees in Commonly Encountered Pigmented Cutaneous Lesions during the COVID-19 Pandemic: A Single Academic Institution Experience. Ann. Diagn. Pathol..

[38] Mosquera-Zamudio A., Launet L., Tabatabaei Z., Parra-Medina R., Colomer A., Moll O., Monteagudo C., Janssen E., Naranjo V. (2023). Deep Learning for Skin Melanocytic Tumors in Whole-Slide Images: A Systematic Review. Cancers.

[39] Sauter D., Lodde G., Nensa F., Schadendorf D., Livingstone E., Kukuk M. (2023). Deep Learning in Computational Dermatopathology of Melanoma: A Technical Literature Review. Comput. Biol. Med..

[40] Cazzato G., Colagrande A., Cimmino A., Arezzo F., Loizzi V., Caporusso C., Marangio M., Foti C., Romita P., Lospalluti L. (2021). Artificial Intelligence in Dermatopathology: New Insights and Perspectives. Dermatopathology.

[41] Zemouri R., Devalland C., Valmary-Degano S., Zerhouni N. (2019). Neural Network: A Future in Pathology?. Ann. Pathol..

[42] Arrastia J.L., Heilenktoetter N., Baguer D.O., Hauberg-Lotte L., Boskamp T., Hetzer S., Duschner N., Schaller J., Maass P. (2021). Deeply Supervised UNet for Semantic Segmentation to Assist Dermatopathological Assessment of Basal Cell Carcinoma. J. Imaging.

[43] Rinck D., Dittmer M., Tinker D., Smith K., Heinecke G. (2023). National Resident Survey in Dermatopathology: The Role of Slide Scanners in Resident Learning. J. Cutan. Pathol..

[44] Decroos F., Springenberg S., Lang T., Paepper M., Zapf A., Metze D., Steinkraus V., Boeer-Auer A. (2021). A Deep Learning Approach for Histopathological Diagnosis of Onychomycosis: Not Inferior to Analogue Diagnosis by Histopathologists. Acta Derm.-Venereol..

[45] Doeleman T., Hondelink L.M., Vermeer M.H., van Dijk M.R., Schrader A.M.R. (2023). Artificial Intelligence in Digital Pathology of Cutaneous Lymphomas: A Review of the Current State and Future Perspectives. Semin. Cancer Biol..

[46] Smith H., Blalock T., Stoff B.K. (2025). Ethics of Artificial Intelligence-Assisted Image Interpretation in Dermatopathology. JAAD Int..

[47] Cazzato G., Massaro A., Colagrande A., Trilli I., Ingravallo G., Casatta N., Lupo C., Ronchi A., Franco R., Maiorano E. (2023). Artificial Intelligence Applied to a First Screening of Naevoid Melanoma: A New Use of Fast Random Forest Algorithm in Dermatopathology. Curr. Oncol..

[48] Jartarkar S.R., Cockerell C.J., Patil A., Kassir M., Babaei M., Weidenthaler-Barth B., Grabbe S., Goldust M. (2023). Artificial Intelligence in Dermatopathology. J. Cosmet. Dermatol..

[49] Polesie S., McKee P.H., Gardner J.M., Gillstedt M., Siarov J., Neittaanmaki N., Paoli J. (2020). Attitudes toward Artificial Intelligence within Dermatopathology: An International Online Survey. Front. Med..

[50] Sturm B., Creytens D., Smits J., Ooms A.H.A.G., Eijken E., Kurpershoek E., Küsters-Vandevelde H.V.N., Wauters C., Blokx W.A.M., van der Laak J.A.W.M. (2022). Computer-Aided Assessment of Melanocytic Lesions by Means of a Mitosis Algorithm. Diagnostics.

[51] Ibraheim M.K., Gupta R., Gardner J.M., Elsensohn A. (2023). Artificial Intelligence in Dermatopathology: An Analysis of Its Practical Application. Dermatopathology.

[52] Shah A., Wahood S., Guermazi D., Brem C.E., Saliba E. (2024). Skin and Syntax: Large Language Models in Dermatopathology. Dermatopathology.

[53] Bao Y., Zhang J., Zhang Q., Chang J., Lu D., Fu Y. (2021). Artificial Intelligence-Aided Recognition of Pathological Characteristics and Subtype Classification of Superficial Perivascular Dermatitis. Front. Med..

[54] Brodsky V., Levine L., Solans E.P., Dola S., Chervony L., Polak S. (2023). Performance of Automated Classification of Diagnostic Entities in Dermatopathology Validated on Multisite Data Representing the Real-World Variability of Pathology Workload. Arch. Pathol. Lab. Med..

[55] Thomas S.M., Lefevre J.G., Baxter G., Hamilton N.A. (2021). Non-Melanoma Skin Cancer Segmentation for Histopathology Dataset. Data Brief.

[56] Mitteldorf C., Tronnier M. (2023). Dermatopathology–Current Status and Development in German-Speaking Dermatology. J. Dtsch. Dermatol. Ges..

[57] Oh Y., Kim H.M., Hong S.W., Shin E., Kim J., Choi Y.J. (2022). Digital Dermatopathology and Its Application to Mohs Micrographic Surgery. Yonsei Med. J..

[58] Gomolin A., Netchiporouk E., Gniadecki R., Litvinov I.V. (2020). Artificial Intelligence Applications in Dermatology: Where Do We Stand?. Front. Med..

[59] Sokolov K., Shpudeiko V. (2022). Dynamics of the Neural Network Accuracy in the Context of Modernization of the Algorithms of Skin Pathology Recognition. Indian J. Dermatol..

[60] Higgins A.D., Dunn R.J., Malikzai O., Ahmadzai M., Gardner J.M., Stoff B.K., McMichael J.R. (2022). Kaposi Sarcoma in Afghanistan: A Case Series from a Tertiary Referral Center. Dermatopathology.

[61] Bertram C.A., Stathonikos N., Donovan T.A., Bartel A., Fuchs-Baumgartinger A., Lipnik K., van Diest P.J., Bonsembiante F., Klopfleisch R. (2022). Validation of Digital Microscopy: Review of Validation Methods and Sources of Bias. Vet. Pathol..

[62] Laggis C.W., Bailey E.E., Novoa R., Stewart C.L., Stoff B., Wanat K.A., Barbieri J., Kovarik C. (2020). Validation of Image Quality and Diagnostic Accuracy Using a Mobile Phone Camera Microscope Adaptor Compared with Glass Slide Review in Teledermatopathology. Am. J. Dermatopathol..

[63] Al-Ali F., Polesie S., Paoli J., Aljasser M., Salah L.A. (2023). Attitudes towards Artificial Intelligence among Dermatologists Working in Saudi Arabia. Dermatol. Pract. Concept..

[64] Reddy S., Shaheed A., Patel R. (2024). Artificial Intelligence in Dermoscopy: Enhancing Diagnosis to Distinguish Benign and Malignant Skin Lesions. Cureus J. Med. Sci..

[65] Kucharski D., Kleczek P., Jaworek-Korjakowska J., Dyduch G., Gorgon M. (2020). Semi-Supervised Nests of Melanocytes Segmentation Method Using Convolutional Autoencoders. Sensors.

[66] Guiter G.E., Sapia S., Wright I.A., Hutchins G.G.A., Arayssi T. (2021). Development of a Remote Online Collaborative Medical School Pathology Curriculum with Clinical Correlations, across Several International Sites, through the COVID-19 Pandemic. Med. Sci. Educ..

[67] Ianni J.D., Soans R.E., Sankarapandian S., Chamarthi R.V., Ayyagari D., Olsen T.G., Bonham M.J., Stavish C.C., Motaparthi K., Cockerell C.J. (2020). Tailored for Real-World: A Whole Slide Image Classification System Validated on Uncurated Multi-Site Data Emulating the Prospective Pathology Workload. Sci. Rep..

[68] Blocker S.J., Cook J., Everitt J.I., Austin W.M., Watts T.L., Mowery Y.M. (2022). Automated Nuclear Segmentation in Head and Neck Squamous Cell Carcinoma Pathology Reveals Relationships between Cytometric Features and ESTIMATE Stromal and Immune Scores. Am. J. Pathol..

[69] Meneveau M.O., Vavolizza R.D., Mohammad A., Kumar P., Manderfield J.T., Callahan C., Lynch K.T., Abbas T., Slingluff C.L., Bekiranov S. (2023). A Step toward Personalized Surgical Decision Making Machine Learning Predicts 1 versus Numerous Melanoma Lymph Node Metastases Using RNA-Sequencing. Ann. Surg..

[70] Evans H., Kimani P.K., Hiller L., Tsang Y.W., Sah S., Gopalakrishnan K., Boyd C., Loughrey M.B., Kelly P.J., Boyle D.P. (2025). What Factors Influence Cellular Pathologists’ Confidence in Case Reporting?. Virchows Arch..

[71] D’Alonzo M., Bozkurt A., Alessi-Fox C., Gill M., Brooks D.H., Rajadhyaksha M., Kose K., Dy J.G. (2021). Semantic Segmentation of Reflectance Confocal Microscopy Mosaics of Pigmented Lesions Using Weak Labels. Sci. Rep..

[72] Brunye T.T., Drew T., Saikia M.J., Kerr K.F., Eguchi M.M., Lee A.C., May C., Elder D.E., Elmore J.G. (2021). Melanoma in the Blink of an Eye: Pathologists’ Rapid Detection, Classification, and Localization of Skin Abnormalities. Vis. Cogn..

[73] Koch E.A.T., Erdmann M., Berking C., Kiesewetter F., Kramer R., Schliep S., Heppt M.V. (2023). Standardized Computer-Assisted Analysis of PRAME Immunoreactivity in Dysplastic Nevi and Superficial Spreading Melanomas. Int. J. Mol. Sci..

[74] Quiohilag K., Caie P., Oniscu A., Brenn T., Harrison D. (2020). The Differential Expression of Micro-RNAs 21, 200c, 204, 205, and 211 in Benign, Dysplastic and Malignant Melanocytic Lesions and Critical Evaluation of Their Role as Diagnostic Biomarkers. Virchows Arch..

[75] Ncube B., Mars M., Scott R.E. (2020). The Need for a Telemedicine Strategy for Botswana? A Scoping Review and Situational Assessment. BMC Health Serv. Res..

[76] van Eck N.J., Waltman L. (2010). Software Survey: VOSviewer, a Computer Program for Bibliometric Mapping. Scientometrics.

[77] Flores J., Misra R., Shah B., Williams Y., Haghighat B., Miranda G., Jani P., Frasier K. (2025). Artificial Intelligence and Machine Learning Transforming Dermatopathology with Diagnosis and Predictive Analytics. Dermis.

[78] Zia S., Yildiz-Aktas I.Z., Zia F., Parwani A.V. (2025). An Update on Applications of Digital Pathology: Primary Diagnosis; Telepathology, Education and Research. Diagn. Pathol..

[79] Elmore J.G., Eguchi M.M., Barnhill R.L., Reisch L.M., Elder D.E., Piepkorn M.W., Brunyé T.T., Radick A.C., Shucard H.L., Knezevich S.R. (2022). Effect of Prior Diagnoses on Dermatopathologists’ Interpretations of Melanocytic Lesions. JAMA Dermatol..

[80] Faghihi A., Fathollahi M., Rajabi R. (2023). Diagnosis of Skin Cancer Using VGG16 and VGG19 Based Transfer Learning Models. Multimed. Tools Appl..

[81] Crowley R.S., Tseytlin E., Jukic D. (2025). ReportTutor—An Intelligent Tutoring System That Uses a Natural Language Interface. AMIA Annu. Symp. Proc..

[82] Tran M., Schmidle P., Guo R.R., Wagner S.J., Koch V., Lupperger V., Novotny B., Murphree D.H., Hardway H.D., D’Amato M. (2025). Generating Dermatopathology Reports from Gigapixel Whole Slide Images with HistoGPT. Nat. Commun..

[83] Lalmalani R.M., Lim C.X.Y., Oh C.C. (2024). Artificial Intelligence in Dermatopathology: A Systematic Review. Clin. Exp. Dermatol..

[84] Kamal R., AlSamhori J.F., Noel A., Qaqish L.N., Jaber L.A., Abujudeh R., Hathal M., Mohammed A.Y., Nashwan A.J. (2025). Transforming Dermatopathology with AI: Addressing Bias, Enhancing Interpretability, and Shaping Future Diagnostics. Dermatol. Rev..

[85] Varnosfaderani S.M., Forouzanfar M. (2024). The Role of AI in Hospitals and Clinics: Transforming Healthcare in the 21st Century. Bioengineering.

[86] Hellmeier F., Brosien K., Eickhoff C., Meyer A. (2024). Beyond One-Time Validation: A Framework for Adaptive Validation of Prognostic and Diagnostic AI-Based Medical Devices. arXiv.

[87] Laohawetwanit T., Gonzalez R.S., Bychkov A. (2024). Learning at a Distance: Results of an International Survey on the Adoption of Virtual Conferences and Whole Slide Imaging by Pathologists. J. Clin. Pathol..

[88] Rohr J.M., Ginnebaugh K., Tuthill M., Pimentel J., Markin R. (2024). Real-Time Telepathology Is Substantially Equivalent to In-Person Intraoperative Frozen Section Diagnosis. Arch. Pathol. Lab. Med..

[89] Matthews G.A., McGenity C., Bansal D., Treanor D. (2024). Public Evidence on AI Products for Digital Pathology. npj Digit. Med..

[90] McGraw D., Mandl K.D. (2021). Privacy Protections to Encourage Use of Health-Relevant Digital Data in a Learning Health System. npj Digit. Med..

[91] Jonnagaddala J., Wong Z.S.-Y. (2025). Privacy Preserving Strategies for Electronic Health Records in the Era of Large Language Models. npj Digit. Med..

[92] Charrière K., Pazart L. (2023). Clinical Evidence Requirements according to the IVDR 2017/746: Practical Tools and References for Underpinning Clinical Evidence of IVD-MDs. Clin. Chem. Lab. Med..

[93] Schwen L.O., Kiehl T.-R., Carvalho R., Zerbe N., Homeyer A. (2023). Digitization of Pathology Labs: A Review of Lessons Learned. Lab. Investig..

[94] Ross A.E., Zhang J., Huang H.-C., Yamashita R., Keim-Malpass J., Simko J.P., DeVries S., Morgan T.M., Souhami L., Dobelbower M.C. (2024). External Validation of a Digital Pathology-Based Multimodal Artificial Intelligence Architecture in the NRG/RTOG 9902 Phase 3 Trial. Eur. Urol. Oncol..

[95] Warraich H.J., Tazbaz T., Califf R.M. (2024). FDA Perspective on the Regulation of Artificial Intelligence in Health Care and Biomedicine. JAMA.

[96] McKay F., Williams B.J., Prestwich G., Bansal D., Hallowell N., Treanor D. (2022). The Ethical Challenges of Artificial Intelligence-Driven Digital Pathology. J. Pathol. Clin. Res..

[97] Chauhan C., Gullapalli R.R. (2021). Ethics of AI in Pathology: Current Paradigms and Emerging Issues. Am. J. Pathol..

[98] BJackson R., Rashidi H.H., Lennerz J.K., de Baca M.E. (2024). Ethical and Regulatory Perspectives on Generative Artificial Intelligence in Pathology. Arch. Pathol. Lab. Med..

[99] Bessen J.L., Alexander M., Foroughi O., Brathwaite R., Baser E., Lee L.C., Perez O., Gustavsen G. (2025). Perspectives on Reducing Barriers to the Adoption of Digital and Computational Pathology Technology by Clinical Labs. Diagnostics.

[100] Clunie D.A. (2020). DICOM Format and Protocol Standardization—A Core Requirement for Digital Pathology Success. Toxicol. Pathol..

[101] Senel E., Bas Y. (2020). Evolution of Telepathology: A Comprehensive Analysis of Global Telepathology Literature between 1986 and 2017. Turk. J. Pathol..

[102] Battazza A., Brasileiro F.C.d.S., Tasaka A.C., Bulla C., Ximenes P.P., Hosomi J.E., da Silva P.F., da Silva L.F., de Moura F.B.C., Rocha N.S. (2024). Integrating Telepathology and Digital Pathology with Artificial Intelligence: An Inevitable Future. Vet. World.

[103] Archila L.R., Smith L., Sihvo H.-K., Westerling-Bui T., Koponen V., O’Sullivan D.M., Camila M., Alexander E.E., Wang Y., Sivasubramaniam P. (2022). Development and Technical Validation of an Artificial Intelligence Model for Quantitative Analysis of Histopathologic Features of Eosinophilic Esophagitis. J. Pathol. Inform..

[104] Lord S.J., Horvath A.R., Sandberg S., Monaghan P.J., M Cobbaert C., Reim M., Tolios A., Mueller R., Bossuyt P.M. (2025). Is This Test Fit-For-Purpose? Principles and a Checklist for Evaluating the Clinical Performance of a Test in the New Era of in Vitro Diagnostic (IVD) Regulation. Crit. Rev. Clin. Lab. Sci..

[105] Song A.H., Jaume G., Williamson D.F.K., Lu M., Vaidya A., Miller T.R., Mahmood F. (2023). Artificial Intelligence for Digital and Computational Pathology. Nat. Rev. Bioeng..

[106] Herington J., McCradden M.D., Creel K., Boellaard R., Jones E., Jha A.K., Rahmim A., Scott P.J.H., Sunderland J., Wahl R.L. (2023). Ethical Considerations for Artificial Intelligence in Medical Imaging: Data Collection, Development, and Evaluation. J. Nucl. Med..

[107] Kothari K., Damoi J.O., Zeizafoun N., Asiimwe P., Glerum K., Bakaleke M.B., Giibwa A., Umphlett M., Marin M.L., Zhang L.P. (2023). Increasing Access to Pathology Services in Low- and Middle-Income Countries through Innovative Use of Telepathology. Surg. Endosc. Other Interv. Tech..

